# The scope for manipulating the polyunsaturated fatty acid content of beef: a review

**DOI:** 10.1186/s40104-015-0026-z

**Published:** 2015-06-24

**Authors:** Payam Vahmani, Cletos Mapiye, Nuria Prieto, David C. Rolland, Tim A. McAllister, Jennifer L. Aalhus, Michael E. R. Dugan

**Affiliations:** Agriculture and Agri-Food Canada, Lacombe Research Centre, 6000 C & E Trail, T4L 1 W1, Lacombe, AB Canada; Department of Animal Sciences, Faculty of AgriSciences, Stellenbosch University, P. Bag X1, Matieland, 7602 South Africa; Department of Agricultural, Food and Nutritional Sciences, University of Alberta, Edmonton, AB T6G 2P5 Canada; Agriculture and Agri-Food Canada, Lethbridge Research Centre, 1st Avenue South 5403, PO Box 3000, T1J 4B1 Lethbridge, AB Canada

**Keywords:** Beef, Biohydrogenation products, Lipids, n-3 fatty acids, Rumenic acid, Vaccenic acid

## Abstract

Since 1950, links between intake of saturated fatty acids and heart disease have led to recommendations to limit consumption of saturated fatty acid-rich foods, including beef. Over this time, changes in food consumption patterns in several countries including Canada and the USA have not led to improvements in health. Instead, the incidence of obesity, type II diabetes and associated diseases have reached epidemic proportions owing in part to replacement of dietary fat with refined carbohydrates. Despite the content of saturated fatty acids in beef, it is also rich in heart healthy *cis*-monounsaturated fatty acids, and can be an important source of long-chain omega-3 (n-3) fatty acids in populations where little or no oily fish is consumed. Beef also contains polyunsaturated fatty acid biohydrogenation products, including vaccenic and rumenic acids, which have been shown to have anticarcinogenic and hypolipidemic properties in cell culture and animal models. Beef can be enriched with these beneficial fatty acids through manipulation of beef cattle diets, which is now more important than ever because of increasing public understanding of the relationships between diet and health. The present review examines recommendations for beef in human diets, the need to recognize the complex nature of beef fat, how cattle diets and management can alter the fatty acid composition of beef, and to what extent content claims are currently possible for beef fatty acids.

## Introduction

Quality and price are key factors considered when consumers purchase beef, and a growing segment of medium- to high-income informed consumers now consider the health implications of beef consumption [1, 2]. The present review will cover recent challenges to long-standing recommendations for beef consumption, the content and composition of beef fat, how beef fat composition can be modified through cattle nutrition and practical considerations when beef with enhanced fatty acid profiles reaches consumers’ plates. The overall objective of the review is to provide some insight into how beef and its constituent fatty acids may now, and in the future, fit into the human diet.

### Revisiting recommendations for beef consumption

Diet effects on human health are often related to several diseases associated with dietary fat, many of which take years to develop, and often result in changes in quality of life and lifespan. Many developed countries suffer from high incidences of obesity, type II diabetes, coronary heart disease (CHD) and cancer. Efforts to examine associated dietary factors, and make recommendations to improve health, have at times fallen short. Recommendations to decrease consumption have been targeted at foods that contain nutrients singled out as culprits, and changes in dietary patterns have in some cases led to even more difficulties. One of the best examples is the recommendation to substitute foods containing saturated fatty acids (SFA) with *trans* fatty acid-rich margarines and refined carbohydrates [3]. Years of recommendations to reduce red meat consumption have not been met by dramatic reductions in the incidence of diseases related to dietary fat; on the contrary, the incidence of obesity and type II diabetes has reached epidemic proportions and has been related to refined carbohydrate consumption [4]. Fatty acids singled out in the Nurses’ Health Study as being problematic for CHD are SFA with chain lengths from 14:0 to 18:0, and a stronger association was found when the polyunsaturated fatty acid (PUFA) to SFA ratio was reduced [5].

The current recommendations to reduce SFA intake are based on the findings from studies in mid-20th century that dietary SFA cause an increase in serum total and LDL-cholesterol and therefore increase the risk of heart disease [6]. These earlier studies overlooked other contributing factors as well as the fact that SFA also increase HDL-cholesterol, which is protective against heart disease. Later studies found that the ratio of total serum cholesterol to HDL-cholesterol is a better indicator of heart disease risk than total or LDL-cholesterol [7]. More recently, many studies have started to question the current dietary recommendations against consuming SFA and revealed that SFA intake is not associated with an increased risk of cardiovascular disease [8–10]. In contrast, substitutions of dietary SFA with refined carbohydrates have resulted in increased obesity and worsen blood lipid profiles by increasing serum triacylglycerol and small, dense LDL particles [11, 12]. Reevaluations are required for the existing dietary recommendations which overstate the health risks of SFA and promote their replacement with alternative nutrients such as refined carbohydrates.

Recently, the dogma that meat consumption should be limited in human diets because of its fatty acid composition has come under close scrutiny [13]. In a recent meta-analysis reviewing 20 studies with more than 1 million subjects, Micha et al. [14] found that consumption of red meats was not associated with higher incidence of CHD and type II diabetes, whereas processed meats were associated with increased incidence of both diseases. The authors suggested that other ingredients (e.g., preservatives such as nitrate) used in processed meats, rather than SFA, contributed to the negative disease outcomes. In Europe, current evidence suggests unprocessed lean red meat is safe to consume as a healthy food choice, and recommendations to limit its consumption in substitution for other protein sources including white meat are not justified [15]. In contrast, in the USA, consumption of both unprocessed and processed red meat still reveal associations with disease outcomes, with a greater hazard ratio for unprocessed red meat [16]. However, not all beef is consumed as unprocessed lean beef. In fact, the most consumed beef product in the USA is hamburger [17] which typically contains 10 to 30% fat. Consequently, it would be prudent to shift research focus away from what to do about the SFA in beef towards how beef fat can be used as a vehicle to deliver health-enhancing fatty acids to consumers.

The Global Burden of Disease Study [18] estimated the contribution of risk factors to disease and disability and identified, among other things, that a low intake of omega-3 (n-3) fatty acids is a concern. The high ratio of omega-6 (n-6) to n-3 fatty acids promotes many diseases from cardiovascular disease and arthritis to cancer, whereas lower ratios have suppressive effects [19]. The n-6 to n-3 ratio of diets during human evolution was estimated to be close to 1:1, whereas current Western diets have ratios close to 15:1 [19]. The great amounts n-6 PUFA in the diet promotes the production of eicosanoids (i.e., prostaglandins, thromboxanes, leukotrienes) formed from arachidonic acid (AA) at the expense of those formed from n-3 fatty acids, specifically eicosapentaenoic acid (EPA) [20]. The disproportionate increase in eicosanoids from AA could result in allergic and inflammatory responses such as increase in platelet aggregation, blood viscosity, vasospasm and vasoconstriction as well as reduced bleeding time [21]. Furthermore, an increased n-6 to n-3 ratio could promote or exacerbate atherogenesis [10]. The balance of n-6 to n-3 fatty acids is therefore an important determinant in reducing the risk of inflammatory and autoimmune disorders such as diabetes, CHD, hypertension, diabetes and arthritis.

In China, the n-6 to n-3 fatty acid ratios of red meat were recently found to range from 6/1 to 23/1 [22]. Unless protected from rumen biohydrogenation, beef naturally contains a low content of n-3 fatty acids including α-linolenic acid (ALA; 18:3n-3) and its long-chain (LC) elongation and desaturation products EPA, docoasapentaenenoic acid (DPA) and docosahexaenoic acid (DHA) [23]. The health benefits ascribed to n-3 fatty acids are mostly related to the LC n-3 s typically found at higher concentrations in fish oil (i.e., EPA and DHA), and efforts have been made to establish dietary reference intakes for these [24]. In contrast, the most common LC n-3 fatty acid in beef is DPA, but it can be readily converted to EPA and DHA [25], and should thus be included when calculating LC n-3 s. Consequently, in populations where little or no oily fish is consumed, beef can still be an important source of LC n-3 fatty acids, particularly when DPA is included [26]. The fact that beef fat can be a source of LC n-3 fatty acids is positive, but again when considering health implications of beef fat, it is important not to narrow the scope of consideration to a few individual or related groups of fatty acids. The complexity of beef fat, and that its effects on human health stems both from individual fatty acids and their combined effects, is under appreciated. Consequently beef producers wanting to improve the health profile of beef require information on which fatty acids would be of interest, and how these can be practically and profitably manipulated by diet to reach levels required to be of benefit to consumers.

### Beef fat content and composition

Beef and meat from other ruminant species are noted for having complex fatty acid profiles compared to meat from monogastric species. Paradoxically, using diet to modify meat composition is much easier in monogastric than ruminant species. Rumen microbes are responsible for both the complexity of beef fatty acid composition and for its lack of resemblance to dietary fatty acid profiles [27]. Rumen microbes produce branched- and odd-chain fatty acids and their precursors, resulting in their deposition in beef lipids. In addition, rumen microbes produce several PUFA biohydrogenation products (PUFA-BHP) including conjugated trienes, conjugated dienes, non-conjugated dienes and monounsaturated fatty acids (MUFA) with a vast array of double bond locations and *cis*/*trans* configurations. Cattle diets typically contain 1-4% lipids, which mainly consist of PUFA including linoleic acid (LA, 18:2n-6) and ALA. When cattle consume feed, dietary lipids are acted upon by microbial lipases in the rumen, releasing mainly free PUFA, which are toxic to rumen microbes [28]. To cope, rumen microbes biohydrogenate PUFA to less toxic SFA, particularly to 18:0, and this process is typically very efficient. Residual PUFA-BHP bypassing the rumen can then be absorbed from the lower gut and incorporated into beef. In a survey of Canadian retail beef (*longissimus lumborum* from strip loin steaks) conducted by Aldai et al. [29], the three most concentrated fatty acids were *cis*9-18:1, 16:0 and 18:0 with concentrations of 38%, 24% and 12%, respectively, constituting 74% of total fatty acids (Table 1). The next eight most concentrated fatty acids (1 to 5% of total fatty acids) accounted for 15.2% of total fatty acids. The next 16 most concentrated fatty acids (0.2 to 1% of total fatty acids) contributed 6.4% to total fatty acids, and the final 60 fatty acids (0.0–0.1% of total fatty acids) accounted for 4.4% of total fatty acids with the majority being PUFA-BHP. Beef analyzed in this survey was collected at retail, and in all likelihood would have been from cattle fed barley grain-based diets (75–90% of dry matter).

**Table 1:** Rank order of fatty acids in Canadian retail strip loin steak (longissiums lumborum)^1^

Rank	Group^2^	Common Name^3^	Fatty Acid^4^	Weight, %	SD^5^
1	MUFA	Oleic	*c*9-18:1	38.08	2.699
2	SFA	Palmitic	16:0	23.95	1.381
3	SFA	Stearic	18:0	12.03	1.667
4	MUFA	Palmitoleic	*c*9-16:1	3.592	0.709
5	PUFA	Linoleic	18:2n-6	2.708	0.786
6	SFA	Myrsitic	14:0	2.289	0.392
7	MUFA	Asclepic	*c*11-18:1	1.847	0.213
8	SFA	Margaric	17:0	1.309	0.298
9	MUFA	Margarolic	*c*9-17:1	1.302	0.298
10	MUFA		*t*10-18:1	1.100	0.688
11	DMA		16:0DMA	1.017	0.422
12	PUFA	Arachidonic	20:4n-6	0.809	0.349
13	MUFA	Myristoleic	*c*9-14:1	0.589	0.202
14	DMA		18:0DMA	0.557	0.222
15	BCFA/SFA		*anteiso*17:0	0.504	0.084
16	MUFA	Vaccenic	*t*11-18:1	0.470	0.164
17	MUFA		*c*13-18:1	0.452	0.121
18	SFA		15:0	0.448	0.103
19	BCFA/SFA		*iso*17:0	0.325	0.053
20	PUFA	ALA	18:3n-3	0.277	0.090
21	PUFA	DPA	22:5n-3	0.274	0.099
22	CLA/PUFA	Rumenic	*c*9,*t*11-18:2	0.273	0.134
23	MUFA		*t*13-*t*14-/*c*6-*c*8-18:1	0.263	0.111
24	PUFA		20:3n-6	0.252	0.082
25	MUFA	Elaidic	*t*9-18:1	0.230	0.069
26	MUFA		*t*15-18:1	0.208	0.051
27	MUFA	Gondoic	*c*11-20:1	0.208	0.061
28	MUFA		*c*11-16:1	0.189	0.060
29	PUFA		*c*9*t*13-/*t*8*c*12-18:2	0.179	0.039
30	PUFA		*c*9*c*15-18:2	0.167	0.034
31	MUFA		*c*7-16:1	0.156	0.020
32	MUFA		*t*6-*t*8-18:1	0.156	0.078
33	MUFA		*c*15-18:1	0.154	0.055
34	BCFA		*iso*16:0	0.136	0.035
35	MUFA		*c*12-18:1	0.122	0.052
36	BCFA/SFA		*anteiso*15:0	0.121	0.036
37	MUFA		*t*12-18:1	0.118	0.045
38	PUFA	Adrenic	22:4n-6	0.114	0.024
39	BCFA/SFA		*iso*18:0	0.113	0.047
40	PUFA	EPA	20:5n-3	0.110	0.063
41	PUFA		*t*11*c*15-18:2	0.101	0.052
42	PUFA		*t*8*c*13-18:2	0.092	0.021
43	MUFA		*t*16-18:1	0.088	0.033
44	SFA		11-cyclohexyl-17:0	0.088	0.027
45	MUFA		*c*13-16:1	0.085	0.024
46	MUFA	Gondoleic	*c*9-20:1	0.084	0.018
47	BCFA/SFA		*iso*15:0	0.084	0.028
48	SFA	Arachidic	20:0	0.080	0.021
49	SFA		19:0	0.077	0.030
50	MUFA		*c*9-15:1	0.076	0.026
51	PUFA+MUFA		*c*9*t*12-18:2/*c*16-18:1	0.062	0.016
52	SFA	Behenic	22:0	0.062	0.018
53	CLA/PUFA	Yurawic	*t*7,*c*9-18:2	0.061	0.027
54	PUFA		20:2n-6	0.051	0.013
55	PUFA	Rumelenic	*c*9*t*11*c*15-18:3	0.049	0.021
56	DMA		18:1DMA	0.047	0.021
57	MUFA		*c*11-17:1	0.043	0.016
58	DMA		16:1DMA	0.043	0.015
59	MUFA		*t*11/*t*12-16:1	0.043	0.008
60	CLA/PUFA		*t*9*c*11-18:2	0.041	0.016
61	CLA/PUFA	Linelaidic	*t*9*t*12-18:2	0.040	0.019
62	MUFA		*c*10-16:1	0.039	0.011
63	PUFA/MUFA		20:3n-3/*c*13-22:1	0.037	0.020
64	MUFA		*c*14-18:1	0.037	0.011
65	MUFA		*c*5-17:1	0.035	0.009
66	MUFA		*c*7-17:1	0.034	0.009
67	PUFA		*t*9*c*12-18:2	0.032	0.014
68	PUFA	GLA	18:3n-6	0.032	0.013
69	SFA	Lauric	12:0	0.032	0.011
70	PUFA	DHA	22:6n-3	0.031	0.018
71	SFA	Lignoceric	24:0	0.030	0.014
72	MUFA		*t*5-18:1	0.027	0.016
73	MUFA		*c*12-16:1	0.026	0.008
74	SFA		21:0	0.023	0.006
75	CLA/PUFA		*t*10,*c*12-18:2	0.011	0.006
76	CLA/PUFA		*c*9,*c*11-18:2	0.011	0.003
77	CLA/PUFA		*t*8,*c*10-18:2	0.011	0.003
78	CLA/PUFA		*c*11,*t*13-18:2	0.009	0.003
79	CLA/PUFA	Mangold's	*t*9,*t*11-18:2	0.009	0.003
80	CLA/PUFA		*t*11,*c*13-18:2	0.008	0.006
81	CLA/PUFA		*t*11,*t*13-18:2	0.005	0.002
82	CLA/PUFA	Mikusch's	*t*10,*t*12-18:2	0.004	0.002
83	CLA/PUFA		*c*12,*t*14-18:2	0.004	0.002
84	CLA/PUFA		*t*12,*c*14-18:2	0.004	0.003
85	CLA/PUFA		*t*7,*t*9-18:2	0.003	0.001
86	CLA/PUFA		*t*12,*t*14-18:2	0.003	0.001
87	CLA/PUFA		*t*8,*t*10-18:2	0.002	0.001

^1^30 steaks collected in summer and 30 in winter [29]

^2^ *SFA* saturated fatty acids, *MUFA* monounsaturated fatty acids, *BCFA* branched-chain fatty acids, *PUFA* polyunsaturated fatty acids, *DMA* dimethyl acetal, *CLA* conjugated linoleic acid

^3^ *ALA* α-linolenic acid, *DPA* docosapentaenoic acid, *EPA* eicosapentaenoic acid, GLA γ-linolenic acid, *DHA* docosahexaenoic acid

^4^ *c cis*, *t trans.* Co-eluting fatty acids on gas chromatogram are separated by a slash (/)

^5^ *SD* standard deviation

Modifying the fat content and composition of beef has been the subject of several reviews, and, in summary, the amount of fat in beef and its composition can be modified primarily by diet and to a lesser extent by gender and genetics [30–34]. Feeding high-grain diets to cattle leads to fatter carcasses and deposition of intramuscular fat (i.e., marbling), a valued attribute in several markets including Japan, the USA and Canada. In contrast to high-grain diets, reducing dietary energy content, through feeding high-forage diets, reduces carcass fatness, decreases intramuscular fat and increases the proportion of PUFA rich phospholipids relative to SFA rich neutral lipids [35]. Feeding high-forage diets can also lead to what is perceived to be a more healthful beef fatty acid profile, but the trade-off with lower energy diets is increased time to market, and the need to be able to source pasture or conserved forage. In addition, increased proportions of forage in the diet can lead to changes in beef palatability [36] such as decreased tenderness because of increased age at finished weights, and the beef may not be as marketable to consumers that value marbling. In countries like Canada and the USA where feedlot finishing on high-grain diets is the norm, finishing on forage-based diets is limited to a small but growing segment of the market [36]. Future expansion of this market will likely depend on whether fatty acid-associated impacts on human health can be scientifically substantiated.

**Fig. 1:**
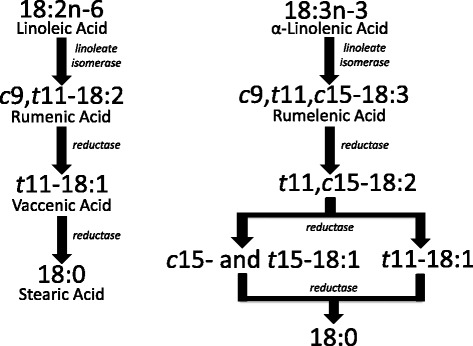
Major pathways for the biohydrogenation of linoleic and α-linolenic acids in the rumen showing isomerization and hydrogenation. Adapted from Harfoot and Hazlewood [39]

The quantity and composition of PUFA-BHP in beef is very much dependent on the supply of PUFA in the diet, and associated dietary and animal factors (e.g., feeding behavior and rumen conditions) which influence the degree of biohydrogenation [37]. In general, pathways used for biohydrogenation of LA and ALA, the major fatty acids in typical cattle diet, are influenced by the forage to concentrate ratio [38]. The most highly characterized pathways for LA and ALA biohydrogenation were elucidated when greater proportions of forage versus concentrate were fed (Fig. 1). Pathways for both LA and ALA are characterized by initial isomerization of the *cis* double bond at carbon 12 to a *trans* double bond at carbon 11 resulting in the production of RA and *cis*9,*trans*11,*cis*15-18:3, respectively [39, 28]. In contrast, when feeding diets with increased amounts of readily fermentable carbohydrate (i.e., high-grain diets), isomerization of the *cis* 9 double bond for LA shifts towards a *trans* double bond at carbon 10 [28], while isomerization of the *cis* 12 double bond of ALA shifts towards a *trans* double bond at carbon 13, resulting in the production of *trans*10, *cis*12-18:2 and *cis*9, *trans*13, *cis*15-18:3, respectively [40]. Following this are rounds of hydrogenation and isomerization leading to *trans* 18:1 isomers (e.g., VA and *trans*13-18:1) and eventually complete hydrogenation to 18:0. However, pathways for the formation of many BHP found in Table 1 have not been established. In addition, new BHP continue to be found. For example, recently *trans*10, *cis*15-18:2 was found to be a BHP of ALA [41], adding one more piece to the puzzle of ALA biohydrogenation pathways. In addition, a great number of BHP of longer chain more highly unsaturated PUFA (e.g., DHA) have also been recently characterized [42].

The fatty acid composition of beef is complex, but concentrations of many fatty acids can be extremely low. Interestingly, the fatty acids in low concentration including many PUFA-BHP have become of interest because of the finding that some can have potent biological activity. The BHP that have been studied the most are VA and RA, which have been shown to have anticarcinogenic and hypolipidemic properties in cell culture and animal models [43–46]. Still, the effects of many PUFA-BHP have not been studied and finding ways to consistently and meaningfully influence their concentrations is of considerable interest.

### Fatty acids of interest and their manipulation by diet

As the effects of fat on ill-health have in part been associated with SFA, logically fat with increased contents of unsaturated fatty acids (UFA), at the expense of SFA, may be more healthful for people to consume. Interestingly, feeding ruminants diets rich in grain are not always associated with greater contents of SFA in meat. In fact, the longer cattle are finished on grain, the greater the delta-9 desaturase activity and conversion of 18:0 to *cis*9-18:1 in beef [47]. Feeding grain-based diets is, however, also associated with increased *trans*10-18:1 deposition [48, 49], and consuming *trans*10-18:1 enriched fats may result in undesirable shifts in plasma cholesterol profiles [50, 51]. Consequently, it would be of importance to determine if the health value of beef enriched with*cis*9-18:1 is still maintained when different proportions of *trans*10-18:1 are present.

Cattle feeding practices most frequently associated with increased proportions of PUFA in beef, particularly n-3 fatty acids, are grazing or feeding preserved forages [36, 52]. From a human nutrition perspective, grazing or feeding cattle forages compared to concentrate is appealing as it reduces the fat content of beef and provides several potential improvements in beef fatty acid composition. Forage finishing can increase the percentage of n-3 fatty acids [53], reduce the n-6 to n-3 ratio, reduce the SFA/PUFA ratio, and increase the percentages of specific PUFA-BHP such as VA and RA [23]. These changes in fatty acid composition may exert protective effects against a number of diseases ranging from cancer to cardiovascular disease [33, 37, 45]. It is thus important to consider if improvements in beef fatty acid composition when including more forage in cattle diets, has any human health benefits over and above those related to reductions in total fat content. In addition, it should be determined if human health benefits are consistent when consuming steak (<10% fat) versus ground beef (10–30% fat). Humans consuming red meat (beef and lamb) from grass or concentrate finished animals were found to have no differences in serum lipids, lipoproteins, triacylglycerols or blood pressure [54]. Interestingly, grass fed beef and lamb were able to increase consumer plasma and platelet LC n-3 PUFA status, leading authors to conclude red meat from grass-fed animals may contribute to dietary intakes of LC n-3 PUFA in populations where red meat is habitually consumed. On the other hand, Wagyu steers finished for an extended period on corn grain versus pasture yielded hamburgers enriched with MUFA and SFA, respectively. Consuming SFA-rich hamburgers decreased serum high-density lipoprotein cholesterol (HDL or “good cholesterol”) in mildly hypercholesterolaemic men [55]. Consuming SFA-rich hamburgers did not, however, change serum low-density lipoprotein (LDL) cholesterol, but did reduce LDL particle diameter, and increased triacylglycerols. Consequently, in the future, it will be important to place these and other results into context when considering which beef or beef products to consume, as these may have differing effects on human health, even when coming from the same animal. For example, as it can be interpreted from studies cited above, lean beef from grass-fed cattle may have a fatty acid profile associated with positive effects on human health, but regular hamburger produced from the same beef may be less favorable in terms of the MUFA/SFA ratio.

Beyond strategies to increase amounts of UFA in beef by feeding forage, a more direct possibility can be through supplementing diets with PUFA rich oils or oilseeds. Nevertheless, this dietary strategy is not without difficulty because of the high efficiency of microbial biohydrogenation of PUFA in the rumen, and the influence of diet on routes of biohydrogenation. Supplementing PUFA in cattle diets has, therefore, frequently led to only minor changes in the PUFA or PUFA-BHP content of beef. For example, Gonzalez et al. [56] found very limited accumulation of PUFA or PUFA-BHP in beef when adding 4.5% sunflower, linseed or soybean oil to a concentrate-based diet, and concluded finding ways to protect PUFA from ruminal biohydrogenation would be an important step to increase the PUFA content of beef. Trying to protect PUFA through feed processing [57] or by chemical treatment (e.g., feeding calcium salts or amides of fatty acid) have met with limited success [58, 59]. Significant ruminal bypass of PUFA has been achieved by Scott and co-workers in Australia when using formaldehyde-treated casein to encapsulate oils [60], and more recently this has been extended to include long-chain n-3 fatty acids [61]. The higher content of PUFA can, however, lead to changes in beef sensory characteristics [62], but oxidative stability has been in part offset by vitamin E supplementation [61]. Encapsulating oils has been noted to be expensive, but the process has also been applied to oilseeds to reduce costs [60]. Encapsulation is certainly an area that could benefit from additional investigation, particularly for n-3 fatty acid rich oilseeds such as flaxseed.

Feeding PUFA rich oils or oilseeds in combination with forage versus concentrate-based diets can also have differing effects on the fatty acid composition of beef. Labrune et al. [63] found increased contents of ALA in beef when feeding flaxseed together with corn, which may have been related to effects of low pH on ruminal lipolysis, the first committed step leading to biohydrogenation [64]. In contrast, when Aldai et al. [65, 66] fed 3% soybean oil in a barley grain-based diet with barley straw as the forage source, there was a preferential accumulation of *trans*10-18:1 in beef at the expense of VA and RA. Supplementing grazing heifers with concentrate fortified with vegetable oils (sunflower or linseed oil) led to remarkable increases in VA and RA in lean beef and adipose tissue [67], but no appreciable increases in VA or RA were found by Kronberg et al. [68] when supplementing steers with flaxseed on pasture. Feeding flaxseed or sunflower seed with either grass-hay or red clover silage-based diets did, however, result in accumulations of VA and RA [69]. In addition, feeding flaxseed supplemented diets resulted in accumulation of BHP specific to ALA, notably *trans* 13/14-18:1, *trans*11,*cis*15-18:2, *trans*11,*cis*13-18:2 and *cis*9,*trans*11,*cis*15-18:3. Accumulations of BHP specific to ALA were reduced when feeding flaxseed together with barley silage compared to grass hay [70].

The quantity and type of forage in cattle diets can be keys to increasing BHP with potential influences on human health. Forage-based diets can promote rumen conditions conducive to VA and RA synthesis. Furthermore, they appear to influence the final step in PUFA biohydrogenation to 18:0, resulting in a differences in BHP outflow from the rumen. In addition to adding forage to the diet, there are some indications that the final step in PUFA biohydrogenation to 18:0 can also be influenced by other means. Long-chain n-3 fatty acids found in fish oil or marine microalgae can inhibit the final step in PUFA hydrogenation to 18:0 [71], but the effects may be variable depending on the composition of the basal diet [72, 73]. In addition, some plant secondary metabolites such as tannins [74], saponins [75] and polyphenol oxidase products [76, 77] have potential to interfere with the final step of ruminal biohydrogenation. In the future, there may also be opportunities to influence ruminal biohydrogenation using direct fed microbials, as several bacterial species with biohydrogenation activity have been indentified [78] and several others have recently been associated with deposition of high and low levels of VA in adipose tissue when feeding steers either flaxseed or sunflower seed [79].

### Genetic and metabolic influences on beef fatty acid composition

As previously mentioned, the amount of intramuscular fat influences the fatty acid composition of beef because of increases in SFA deposition as total fat increases [35]. Beyond this, fatty acid composition has been noted to have low to moderate heritability [47, 80, 81, 30], but efforts to use genetic selection to improve beef fatty acid composition have been limited for a number of reasons [30]. First, fatty acid composition is not a single trait and it is not clear at present the type or number of fatty acids or their derived parameters that should be included as criteria in a breeding program. Second, if the PUFA/SFA ratio is one criterion for selection, the favorable correlation with reduced fatness suggests that an improved PUFA/SFA ratio can probably be more easily obtained by selecting for lower fatness versus direct selection for individual fatty acids. Third, measuring fatty acid composition on a large number of animals for breeding value estimation would be expensive using conventional techniques (i.e., gas chromatography). Beyond conventional breeding strategies, however, recent developments in genomic technologies have provided opportunities for marker-assisted selection. Single nucleotide polymorphisms (SNPs) have been found for a number of candidate genes involved in fatty acid metabolism [82–85]. A 54 k single nucleotide polymorphism (SNP) chip has also now been used to investigate possibilities for marker-assisted selection of multiple traits from basic meat quality to nutritional composition including mineral and fatty acid composition [86]. Greater advances in the area may thus be on offer with >54 k chips, and with this, the potential for finding quantitative trait loci (QTL) and identify specific genes associated with variation in fatty acid composition. Rapid and low cost fatty acid analysis is, however, needed to match the pace of development in genomic technologies (higher speed genotyping at lower and lower costs). Along this line, the use of newer non-destructive technologies, such as near infra-red spectroscopy (NIRS), to measure beef fatty acid composition have shown promise [87–89], with the ability to predict the content of a number of fatty acids in beef fat related to human health. Further study of fatty acid synthesis and metabolism in beef cattle at the fundamental biochemical and molecular levels is also required to help explain breed, inter-animal and tissue (e.g., adipose vs. muscle) differences. Understanding these differences would then allow opportunities to identify physiological and nutritional factors that influence gene expression and enzyme activity, providing additional avenues to improve beef fatty acid composition [90].

### Regulations, recommendations and delivery of PUFA and PUFA-BHP in beef

In the past 10–15 years, manipulating the PUFA and PUFA-BHP content of beef has been intensively investigated. However, to be of practical importance for the industry (1) the profiles and concentrations needed for health benefits must be defined (2) requirements established and (3) source and health claims generated [37]. More importantly, consumers should be informed about potential health benefits of consuming beef products enriched with PUFA or PUFA-BHP. How nutrient source and health claims are handled vary from country to country, and in some countries basic nutritional labelling of foods is not even required [91]. Some countries have an agency that regulates the use of health claims (for example, Health Canada in Canada, the Food and Drug Administration in the USA, The Ministry of Health, Labour, and Welfare in Japan, the Korean Food and Drug Administration, the State Food and Drug Administration in China, the Food Control Department in Singapore and the Department of Health in South Africa). Historically, some governments permitted health claims but left it up to private interests to regulate their use (United Kingdom and Sweden). Other countries have decided to cooperatively develop regulations together on health and nutrition claims (e.g., the European Union, Australia and New Zealand). Given the between country differences, the present review focuses on fatty acid claims permitted in Canada, USA and the European Union as examples of what similarities and differences exsist between countries even when claims are permitted.

Currently, for fatty acids of greatest interest (i.e.,n-3 and certain PUFA-BHP), source claims can only be made for n-3 fatty acids in Canada, the USA and the European Union. In Canada, a source of n-3 fatty acids has to have at least 300 mg of total n-3 fatty acids per 100 g serving [92]. In the USA, foods with ≥ 160 mg or ≥ 320 mg ALA can be referred to as a “source” or “rich” in ALA, and no claims can be made for EPA or DHA [93]. In the European Union, foods with 300 mg ALA or 40 mg combined EPA and DHA per 100 g can be labeled as a source of n-3 fatty acids, and foods with 600 mg ALA or 80 mg combined EPA and DHA per 100 g can be labeled as rich in n-3 fatty acids [94]. Meeting the label requirements for different markets thus require different production strategies.

Irrespective of requirements for each country, it has been difficult to achieve target amounts of n-3 fatty acids in lean beef [95]. LaBrune et al. [63] reached a high of 2.1% ALA in lean beef when feeding flaxseed in a corn-based diet. Estimating 4–6% fat in lean beef, a yield of 84–126 mg ALA per 100 g serving would have been achieved. In pork chops from pigs fed flaxseed, inclusion of some level of external trim fat is required to meet labeling requirements for an n-3 source claim in Canada [96]. Consequently, with slightly more marbling fat or inclusion of a small amount of external trim fat in a serving, the beef from LaBrune et al. [63] may have been able to reach a source claim in the USA (i.e., 160 mg of ALA per serving). Although most studies have not been able to exceed 2% ALA in lean beef fatty acids even when feeding supplemental flaxseed [97–102, 69, 70, 103], there may still be potential to achieve claims in ground beef and further processed beef products. For example, Nassu et al. [70] estimated regular (30% fat) ground beef from flaxseed fed cows would have contained as much as 339 mg of total n-3 fatty acids per 4 oz (114 g) serving. On the other hand, in Europe, meeting a source claim for combined EPA and DHA would be very difficult without feeding some form of protected long-chain n-3 fatty acids [61, 23], although some success has been achieved when feeding fish meal as opposed to fish oil [104]. Again, there would be some potential for a source claim in the EU for ground beef when feeding flaxseed, but only if DPA could be included with EPA and DHA as a long-chain n-3 fatty acid, as is the case in Australia and New Zealand [105], and South Africa [106]. For example, Nassu et al. [70] estimated regular (30% fat) ground beef from flaxseed fed cows would contain as much as 39.4 mg EPA + DPA + DHA per 4 oz (114 g) serving. Consequently, there are definite possibilities to produce beef capable of entering the n-3 fatty acid-enriched market. However, the economic feasibility will depend on balancing the consumer’s willingness to pay for the enhanced nutritional attributes versus the cost of production [107, 108]. Hence research geared to reliably and cost effectively enhance fatty acid composition is of relevance.

Beyond n-3 fatty acids, the greatest potential for enriching beef with healthful fatty acids is likely with PUFA-BHP, specifically VA and CLA. In 2005, Dhiman et al. [109] estimated a serving (100 g) of beef steak enriched with CLA would provide about 41 mg of CLA, and taken together with other foods (mainly whole milk and cheese) would exceed the 300 mg of CLA per day calculated to be required to reduce the incidence of cancer in humans [110]. The major isomer of CLA is RA with its precursor, VA, having a 19% conversion efficiency in humans [111]. Based on RA equivalents (RA + 0.19*VA), Sofi et al. [112] found humans consuming cheese providing 203 mg RA equivalents per day elicited favorable changes in atherosclerotic markers. Consumption of between 200 and 300 mg RA equivalents, therefore, seems to be a reasonable estimate for the amount of RA needed to elicit positive effects on human health. Consumption of 200–300 mg RA is considerably less than the 3.4 g per day thought to be required to induce a reduction in body fat [113]. Recent results indicate enriched beef may be able to provide substantially more than the 41 mg CLA per day as estimated by Dhiman et al. [109]. Noci et al. [67] supplemented pastured heifers with sunflower oil yielding ~127 mg RA equivalents per 100 g serving of lean beef. Mapiye et al. [69] feeding rolled flaxseed together with red clover silage also produced lean beef with 173 mg RA equivalents per 100 g serving. Using a similar feeding strategy in a follow up study Mapiye et al. [102] only produced 29 mg RA equivalents in lean beef, but from the same experiment, Turner et al. [114] produced hamburgers made with 20% perirenal fat that contained 319 mg RA equivalents per 100 g serving. In addition, these hamburgers contained 49 mg of *cis*9,*trans*11,*cis*15-18:3 and 224 mg of its precursor *trans*11,*cis*15-18:2. Such alterations in fatty acid profile could add further value to the hamburgers if the health effects of these fatty acids are similar to plant-derived conjugated linolenic acid isomers [115, 116]. However, for any of the PUFA-BHP, their health value in beef still needs to be recognized by regulatory authorities, and recommended intakes need to be defined before requirements for enrichment levels can be established. Subsequent to this, studies would still be necessary to define/refine cost effective production strategies to produce beef with required and consistent enrichments of various PUFA-BHP.

## Conclusions

1) Early investigations linking SFA intake with diet-related diseases in humans led to recommendations that consumption of red meat, including beef, should be reduced. Changes in dietary patterns that ensued did not lead to improvements in health, but instead led to increases in prevalence of obesity and type II diabetes. 2) Recommendations to reduce red meat intake still persist, but some recent evidence indicates this may not always be justified. 3) Beef can be an important source of LC n-3 fatty acids, and the potential to increase these should be a research priority. Research should be at the feeding level, but also at the fundamental level in understanding and potentially capitalizing on differences in pathways for LC n-3 fatty acid synthesis. In addition, recent evidence suggests the recognition of DPA, as well as EPA and DHA, as a dietary source of LC n-3 fatty acids, may be justified. 4) The complexity of beef fat composition may also have untapped potential in the form of PUFA-BHP. Although, the concentration of many PUFA-BHP can be quite low, methods to selectively increase or decrease these fatty acids have not been thoroughly investigated. 5) All told, given the complexity and differences in fatty acid composition within beef carcass fat depots (e.g., intramuscular fat versus subcutaneous fat) and the differences in beef product fat content and source, recommendations for beef consumption should not be generalized. Rather these recommendations need to evolve as our knowledge of individual and combined health effects of beef fatty acids develop.

The health effects of some fatty acids (e.g., n-3) are known, and recommended intakes have been defined, leading to opportunities to make enrichment claims in beef. For other fatty acids (e.g., PUFA-BHP), the need for these in the human diet still needs to be accepted by regulatory authorities and source claims developed. Once source claims are possible, production of PUFA-BHP enriched beef could move from proof of concept towards development/refinement of economically feasible production strategies.

## Abbreviations

ALA: α-linolenic acid
CHD: Coronary heart disease
DPA: Docoasapentaenenoic acid
DHA: Docosahexaenoic acid
EPA: Eicosapentaenoic acid
HDL: High density lipoprotein
LA: Linoleic acid
LC: Long-chain
LDL: Low density lipoprotein
MUFA: Monounsaturated fatty acids
PUFA: Polyunsaturated fatty acid
PUFA-BHP: PUFA biohydrogenation products
RA: Rumenic acid
SFA: Saturated fatty acids
UFA: Unsaturated fatty acids
VA: Vaccenic acid
